# Quantification of the Monomer Compositions of Poly(3-hydroxybutyrate-co-3-hydroxyvalerate) and Poly(3-hydroxyvalerate) by Alkaline Hydrolysis and Using High-Performance Liquid Chromatography

**DOI:** 10.3390/bioengineering10050618

**Published:** 2023-05-20

**Authors:** Kyo Saito, M. Venkateswar Reddy, Omprakash Sarkar, A. Naresh Kumar, DuBok Choi, Young-Cheol Chang

**Affiliations:** 1 Course of Chemical and Biological Engineering, Division of Sustainable and Environmental Engineering, Muroran Institute of Technology, 27-1 Mizumoto, Muroran 050-8585, Japan; 21041026@mmm.muroran-it.ac.jp; 2 Department of Civil and Environmental Engineering, Colorado State University, Fort Collins, CO 80523, USA; mvr_234@yahoo.co.in; 3 Department of Civil, Environmental and Natural Resources Engineering, Luleå University of Technology, 97187 Luleå, Sweden; ommsarkar@gmail.com; 4 Department of Environmental Science and Technology, University of Maryland, College Park, MD 20742, USA; 5 Faculty of Advanced Industry Convergence, Chosun University, Kwangju 61452, Republic of Korea

**Keywords:** chromatography, crotonic acid, homopolymer, copolymer, polyhydroxyalkanoate, poly(3-hydroxyvalerate)

## Abstract

With the growing interest in bioplastics, there is an urgent need to develop rapid analysis methods linked to production technology development. This study focused on the production of a commercially non-available homopolymer, poly(3-hydroxyvalerate) (P(3HV)), and a commercially available copolymer, poly(3-hydroxybutyrate-co-3-hydroxyvalerate) (P(3HB-*co*-3HV)), through fermentation using two different bacterial strains. The bacteria *Chromobacterium violaceum* and *Bacillus* sp. CYR1 were used to produce P(3HV) and P(3HB-*co*-3HV), respectively. The bacterium *Bacillus* sp. CYR1 produced 415 mg/L of P(3HB-*co*-3HV) when incubated with acetic acid and valeric acid as the carbon sources, whereas the bacterium *C. violaceum* produced 0.198 g of P(3HV)/g dry biomass when incubated with sodium valerate as the carbon source. Additionally, we developed a fast, simple, and inexpensive method to quantify P(3HV) and P(3HB-*co*-3HV) using high-performance liquid chromatography (HPLC). As the alkaline decomposition of P(3HB-*co*-3HV) releases 2-butenoic acid (2BE) and 2-pentenoic acid (2PE), we were able to determine the concentration using HPLC. Moreover, calibration curves were prepared using standard 2BE and 2PE, along with sample 2BE and 2PE produced by the alkaline decomposition of poly(3-hydroxybutyrate) and P(3HV), respectively. Finally, the HPLC results obtained by our new method were compared using gas chromatography (GC) analysis.

## 1. Introduction

The replacement of synthetic plastics by bioplastics is gaining attention; hence, polyhydroxyalkanoates (PHAs) are increasingly sought after due to their desirable properties, such as biodegradability and biocompatibility [1]. Poly(3-hydroxybutyrate) (P(3HB)) is among the PHAs with the highest commercial production, and many industries commercially produce P(3HB) in large scale. In 1925, the French scientist Lemoigne first reported P3HB production in *Bacillus megaterium* [2]. Since then, bacteria such as *B. cereus*, *B. subtilis*, *Cupriavidus necator*, *Pseudomonas putida*, and *Rhodococcus* sp. have been used to produce P(3HB) [2,3,4,5,6,7,8]. P(3HB) accumulation occurs in bacteria as a natural stress response. Therefore, if it is possible to create stress conditions in vitro by restricting nutrients, the production of P(3HB) from bacteria can be enabled. Apart from P(3HB), the copolymer poly(3-hydroxybutyrate-*co*-3-hydroxyvalerate) (P(3HB-*co*-3HV)) has great potential due to its mechanical properties similar to those of polypropylene and low-density polyethylene (LDPE) [9]. To date, P(3HB-*co*-3HV) has been produced in microorganisms such as *B. aryabhattai*, *Cupriavidus necator*, *B. cereus*, *Methylobacterium organophilum*, *Burkholderis* sp., *Corynebacterium glutamicum*, *Massilia haematophila*, *Pseudomonas denitrificans*, and *Hydrogenophaga pseudoflava* [10,11,12,13,14]. Poly(3-hydroxyvalerate) (P(3HV)) is a homopolymer that is synthesised by *Chromobacterium violaceum*, with yields of 62% P(3HV) and 39 g/L dry cell weight reported by Steinbuchel and Schmack (1995) [15,16]. Among other bacteria, *Cupriavidus necator* is able to produce 5 wt% P(3HV) [17], whereas *Cupriavidus necator* accumulates 79% PHA, which is a copolymer of P(3HB-co-3HV) with 12% 3HV content [18]. Improvements have been made to the methods for the extraction and quantification of P(3HB). Initially, P(3HB) was extracted from dried cells using chloroform, followed by crystallisation using a poor solvent, ethanol, and quantification by a gravimetric method [19]. A poor solvent is one in which the solute (PHA) precipitates. Subsequently, a method was developed in which dried cells were lysed with hypochlorous acid and quantified by nephelometric analysis [20]. Later, without extracting the P(3HB) from bacteria, dry biomass was treated with concentrated sulfuric acid, and the P3HB inside the cell was converted into crotonic acid, which was subsequently quantified by ultraviolet absorption [21]. A gas chromatography (GC) method was developed for quantifying the methyl-3-hydroxybutyrate produced by the methanolysis of dried cells in the presence of sulfuric acid and chloroform [22]. P(3HB) granules present in the live bacterial cells were then observed under a microscope by staining with Nile red [23]. As P(3HB) granules are closely surrounded by proteins, lipids, and enzymes such as P(3HB) synthase and P(3HB) depolymerase, pretreatment is required to remove these components and produce a pure product [24,25]. P(3HB) quantification by high-performance liquid chromatography (HPLC) analysis (by measuring the crotonic acid) after acid hydrolysis has recently become widely used [21]. The alkaline decomposition of P(3HB) and P(3HV) results in the production of 2BE and 2PE, respectively. During pretreatment, P(3HB) and P(3HV) are hydrolysed by NaOH to produce monomers. After that, the hydroxide ion, which is a nucleophile under alkaline conditions, is thought to undergo a nucleophilic reaction with the α proton. Elimination and substitution reactions then occur to form 2-alkenoic acids. At this time, it is considered that the longer alkyl side chain bonds to the α carbon, thereby increasing the electron density and lowering the nucleophilicity [26]. Therefore, it is believed that the length of the alkyl side chain of the monomer is the cause of the difference in absorption sensitivity. From this, it was shown that the production ratio α of 2BE from P(3HB) and the production ratio β of 2PE from P(3HV) are constant. Duvigneau et al. reported that acidic hydrolysis of the heteropolymers resulted in monomers that had overlapping UV spectra and were therefore impossible to differentiate [27]. Duvigneau et al. also reported that GC methods provide more detailed and precise measurements. For reasons of accuracy, many research groups use these analysis methods, but toxic solvents such as chloroform and dichloromethane are required. A good alternative is HPLC, because different detection systems, such as UV detectors, can be used for analysis. In addition, in comparison to GC methods, LC methods are less time-consuming, given sample preparation. Further, samples for LC can be taken directly from the culture broth, whereas GC measurements need dried biomass. Most of the available detection methods have focused on P(3HB) [28], while few methods have focused on the monomer content in copolymers such as P(3HB-*co*-3HV), and no methods have focused on P(3HV) analysis. Hence, this study aims to produce a commercially non-available homopolymer, P(3HV), and a commercially available copolymer, P(3HB-*co*-3HV), using the bacteria *Chromobacterium violaceum* and *Bacillus* sp. CYR1, respectively. The other objective is to develop a fast, simple, and inexpensive method to quantify P(3HV) and P(3HB-*co*-3HV) using HPLC through the alkaline decomposition method. P(3HB-*co*-3HV) was purchased from Sigma-Aldrich (Cat# 403121-10G) and was also produced in this study, but P(3HV) was only produced in this study, since it is commercially non-available. The alkaline decomposition of P(3HB-*co*-3HV) releases 2-butenoic acid (2BE) and 2-pentenoic acid (2PE). The concentrations of these compounds were measured using HPLC. Moreover, calibration curves were prepared using standard 2BE and 2PE, along with samples of 2BE and 2PE produced by the alkaline decomposition of P(3HB) and P(3HV). Finally, the HPLC results obtained via our method were compared using GC analysis.

## 2. Materials and Methods

### 2.1. P(3HB-co-3HV) Production

The bacterium *Bacillus* sp. CYR1 (DDBJ accession number LC049103) previously isolated in our laboratory was used to produce the copolymer P(3HB-*co*-3HV) [6]. A preculture was prepared by incubating the CYR1 strain in Nutrient Broth (NB) medium. The pH of the NB medium was adjusted to 7.0 before sterilisation. After sterilisation, the CYR1 strain was added to Erlenmeyer flasks containing NB medium and incubated under shaking at 100 rpm and 30 °C for 8 h. To produce P(3HB-*co*-3HV), 8% (*v*/*v*) preculture was added to 300 mL of mineral salt (MS) medium containing acetic acid (10 g/L) and valeric acid (10 g/L) in 1 L Erlenmeyer flasks. The flasks were subsequently incubated under shaking at 100 rpm and 30 °C for 108 h. Experiments involving P(3HB-*co*-3HV) were conducted as described by Reddy et al. [6]. The extraction of P(3HB-*co*-3HV) was carried out as described in Section 2.3. 

### 2.2. P(3HV) Production

The bacterium *Chromobacterium violaceum* (NBRC 12614) was used to produce the homopolymer P(3HV) as detailed previously [15,16]. A preculture was prepared in NB medium containing 0.8 g of Difco™ Nutrient Broth and 0.5 g of sodium valerate (BLDpharm, Cincinnati, OH, USA). To produce P(3HV), 10 mL of preculture was added to 300 mL of mineral salt (MS) medium in 1 L Erlenmeyer flasks. The MS medium had the following composition per litre: 1.0 g K_2_HPO_4_, 0.05 g NaCl, 0.2 g MgSO_4_·7H_2_O, 0.05 g CaCl_2_, 0.0083 g FeCl_3_·6H_2_O, 0.014 g MnCl_2_·4H_2_O, 0.017 g NaMoO_4_·2H_2_O, and 0.001 g ZnCl_2_. Initially, 0.22 g of sodium valerate was added as a carbon source, and the pH was adjusted to 7.0 with 6 N NaOH. Cultivation was performed at 100 rpm for 24 h at 30 °C. Sodium valerate (0.22 g each time) was supplemented after 24 and 48 h of incubation, and the pH was adjusted to 7.5. After 78 h of cultivation, the culture liquid was centrifuged at 8000× *g* for 10 min at 4 °C, the supernatant was discarded, and the resulting pellet was dried for 12 h at 50 °C. P(3HV) extraction was subsequently carried out as described in Section 2.3. Bacterial growth was monitored by measuring the optical density at 600 nm using a UV spectrometer (UV-1800, Shimadzu, Japan). At each sampling interval, 5 mL of sample was collected. Experiments were performed in triplicate, and the data are presented as the mean ± standard deviation.

### 2.3. Extraction of P(3HB-co-3HV) and P(3HV)

P(3HB-*co*-3HV) and P(3HV) were extracted using the Soxhlet extraction method as previously described [29]. The dried pellet was placed in an extraction thimble, CHCl_3_ was added in the round-bottom flask, and Soxhlet extraction was carried out at 80 °C for 48 h. 

#### 2.3.1. Alkaline Decomposition of Homopolymers P(3HB) and P(3HV)

To generate 2BE and 2PE samples, alkaline decomposition was carried out by incubating a known amount of P(3HB) or P(3HV) with 4 mL of NaOH (5 M) at 120 °C for 30 min in screw-cap tubes. After cooling, the pH was adjusted to 3 by adding 6 M HCL, and the volume was adjusted to 10 mL with ultrapure water [26]. The reaction sample was filtered through a hydrophobic PTFE membrane (GLC, diameter 13 mm, pore size 0.22 µm) and was analysed via HPLC [26]. P(3HB) was purchased from Sigma-Aldrich (Burlington, MA, USA), whereas P(3HV) was produced in this study using *C. violaceum* according to the methods described in Steinbüchel et al. since it is not commercially available [15,16].

#### 2.3.2. Alkaline Decomposition of Copolymer P(3HB-*co*-3HV)

As P(3HB-*co*-3HV) is a copolymer of 3-hydroxybutyrate (3HB) and 3-hydroxyvalerate (3HV), 2BE and 2PE are produced by the alkaline decomposition of P(3HB-*co*-3HV). For this, we used the equation P(3HB) = α × 2BE and P(3HV) = β × 2PE, because the production ratio α of 2BE from P(3HB) and the production ratio β of 2PE from P(3HV) are constant in the reaction under the same decomposition conditions. These production ratios were determined by preparing calibration curves using 2BE and 2PE standards, which were purchased from Kanto Chemical and Tokyo Chemical Industry, respectively. Calibration curves of 2BE and 2PE samples produced by the alkaline decomposition of P(3HB) and P(3HV), respectively, were also prepared. The generated calibration curves were then used to quantify P(3HB) and P(3HV). P(3HB-*co*-3HV) was quantified from the combined amount of P(3HB) and P(3HV). P(3HB-*co*-3HV) copolymer was purchased from Sigma-Aldrich (containing 12% PHV) and also produced using *Bacillus* sp. CYR1. P(3HB-*co*-3HV) was pretreated using the alkaline decomposition method, and the resulting 2BE and 2PE were analysed via HPLC.

### 2.4. Analysis

#### 2.4.1. HPLC Analysis

##### Calibration Curve Preparation Using Standard 2BE, 2PE, and 3HB

Different concentrations (0, 25, 50, 75, 100 mg/L) of 2BE (Kanto Chemical, Tokyo, Japan, Cat# 07465-00, purity > 99%) and 2PE (Tokyo Chemical Industry, Tokyo, Japan, Cat# P0345, purity > 96%) standards were prepared by diluting with distilled water using a test tube mixer. Solutions of 3HB with different concentrations were also prepared (0 to 10 mg/L). Known amounts of standard samples, P(3HB) (Sigma-Aldrich), and P(3HV) were taken in screwcap test tubes, and alkaline decomposition pretreatment and HPLC analysis were performed to create calibration curves. Since there is no standard reagent for P(3HV), P(3HV) production experiments were performed using a *Chromobacterium violaceum* strain (NBRC 12614), referring to Section 2.2, and a calibration curve was created using P(3HV) extracted from cells. Subsequently, HPLC (Shimadzu, Kyoto, Japan) was used to analyse these standards. For this, 5 µL of each standard was injected into the column (SHIMADZU, SCR-102H, Kyoto, Japan), and the absorbance was measured at a wavelength of 210 nm using a UV–VIS detector (SHIMADZU, SPD-10AV, Kyoto, Japan). Filtered and degassed 5 mmol/L perchloric acid was used as the mobile phase at a flow rate of 1.5 mL/min. The column temperature was maintained at 45 °C. Calibration curves were prepared with known concentrations, and the absorption sensitivity (peak area/concentration) was determined. As 3HV is not commercially available, a calibration curve was not prepared. Calibration curves of 2BE and 2PE samples produced by the alkaline decomposition of P(3HB) and P(3HV), respectively, were prepared. The generated calibration curves (Section 3.2.2) were then used to quantify P(3HB) and P(3HV). 

#### 2.4.2. GC Analysis

P(3HB-*co*-3HV) produced by the *Bacillus* sp. CYR1 strain was quantified by the alkaline decomposition method using HPLC and GC analysis. For GC analysis, samples were pretreated by methanolysis as described by Tamang et al. [30]. The reaction mixture containing P(3HB-*co*-3HV) or cell pellet, 2 mL of 3% sulfuric acid in 100 mL methanol, 50 mg of benzoic acid (as an internal standard), and 2 mL of chloroform was transferred into screw-cap test tubes and incubated at 100 °C for 3.5 h. Through this reaction, each copolymer composition of P(3HB-*co*-3HV) was methyl-esterified. After cooling to room temperature, 1 mL of ultrapure water was added, and the reaction mixture was stirred with a test tube mixer. The mixture was allowed to settle for 10 min for phase separation. The lower organic phase was separated and dehydrated with 0.5 g of sodium sulfate, and subsequently, 1 µL of the organic phase was injected into a gas chromatograph (GC-2014, Shimadzu, Kyoto, Japan) equipped with a flame ionisation detector (FID) and a Zebron ZB-FFAP column (Phenomenex Inc. CA, USA) with a length of 30 m and an internal diameter of 0.32 mm. The injection temperature was 180 °C, and the column temperature was set at 145 °C. The injection mode was split into a 1:48 ratio, and helium was used as a carrier gas at a flow rate of 2.36 mL/min. The detector temperature was set to 260 °C. A calibration curve was created using standard P(3HB-*co*-3HV) (Sigma-Aldrich), and 3HB and 3HV were quantified by the internal standard method.

## 3. Results and Discussion

### 3.1. Bacterial Growth and Polymer Production

**Figure 1:**
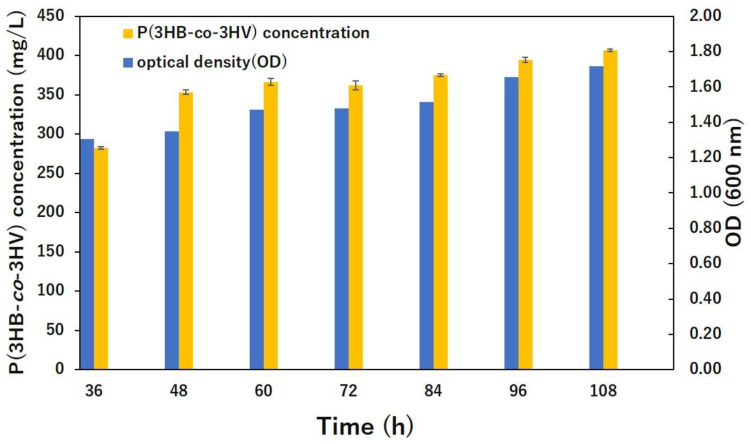
Bacterial growth (OD_600_ values on the right-hand-side Y-axis) and P(3HB-*co*-3HV) (concentration values on the left-hand-side Y-axis) measurement in *Bacillus* sp. CYR1 strain. Some error bars are hidden in the figure as the deviation was too small.

The OD_600_ value and P(3HB-*co*-3HV) concentration showed a correlation, making it possible to quantify P(3HB-*co*-3HV) in dried cells (Figure 1). The bacterial growth and pH increased during P(3HB-*co*-3HV) production. From hour 36 to 108, the bacteria showed incremental growth, reaching the peak (OD_600_ 1.82) at hour 108. Similarly, P(3HB-*co*-3HV) levels also increased from hour 36, reaching the highest level (415 mg/L) at hour 108. The medium pH at hour 36 was 7, and the pH slowly increased to 8 by hour 108.

**Figure 2:**
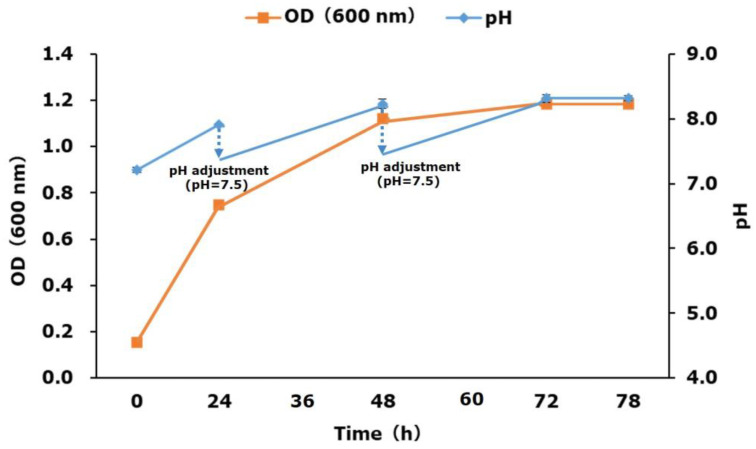
Growth curve (OD_600_ values on the left-hand-side Y-axis) and pH change (values on the right-hand-side Y-axis) over time during P(3HV) production experiments. Error bars are hidden in the figure as the deviation was too small.

Regarding P(3HV) production, we observed incremental growth of bacteria (*C. violaceum*) from hour 0 to 78, reaching the peak (OD_600_ 1.2) at hour 78 (Figure 2). The medium pH at hour 0 was 7.2. Afterwards, the pH gradually decreased and was adjusted to 7.5 at different time periods, finally increasing to 8.2 at hour 78. Since *C. violaceum* uses sodium valerate as a carbon source, an increase in pH was observed as the sodium valerate was consumed. Limited publications are available on P(3HV) production. Steinbuchel and Schmack (1995) used *C. violaceum* DSM 30191 for P(3HV) production [19]. They performed experiments in 10 L and 350 L reactors by keeping the pH of the medium constant at 7.5 during the first phase and at 8.0 during the second phase. They used valeric acid as a carbon source at 3 and 5 g/L and supplemented it with complex nutrients such as peptone, nutrient broth, tryptone, yeast extract, or malt extract. They performed lyophilisation to dry the biomass, and P(3HV) was determined by GC analysis, achieving yields of 39 g/L dry cell weight and 62% PHA. In another study, Steinbuchel et al. (1993) reduced the concentration of ammonium chloride in the medium and used nutrient broth (0.8%, *w*/*v*) as a complex medium [15]. They used valerate and different carbon sources such as fructose, gluconate, propionate, hexanoate, and heptanoate to achieve 43% PHA.

### 3.2. HPLC Results

#### 3.2.1. Calibration Curve Results of 2BE, 2PE, and 3HB Standards

**Figure 3:**
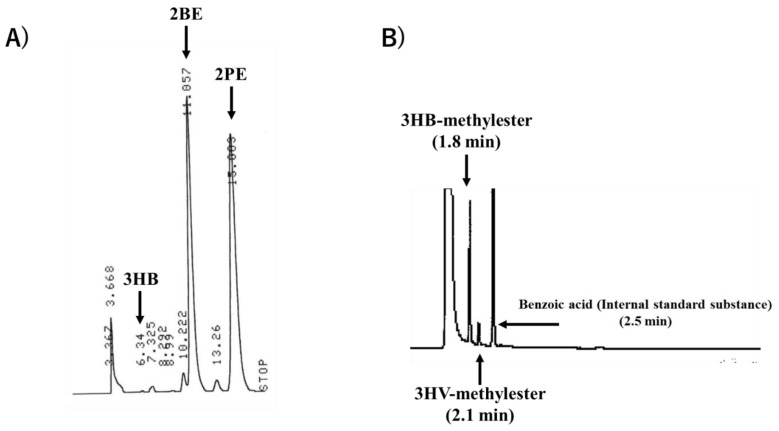
(**A**) HPLC chromatogram of 2BE (retention time: 11.1 min), 2PE (retention time: 15 min), and 3HB (6.3 min). (**B**) GC chromatogram of 3HB-methylester (retention time: 1.8 min), 3HV-methylester (retention time: 2.1 min), and benzoic acid (retention time: 2.5 min). The calibration curve of the P(3HB-*co*-3HV) standard was corrected based on a standard internal substance (benzoic acid) and used to determine the 3HB and 3HV content. The P(3HB-*co*-3HV) standard was obtained from Sigma-Aldrich.

The retention times of the 2BE, 2PE, and 3HB standards were 11.1 min, 15.0 min, and 6.3 min, respectively (Figure 3A). From the obtained absorption sensitivity, we found that 2BE and 2PE, which have double bonds in their structures, can be detected with higher sensitivity than 3HB (Satoh et al., 2016 [31]; Duvigneau et al., 2021 [27]) (Figure 3A). 

At each sampling interval, 5 mL of sample was collected and 0.66 g of sodium valerate was added to the culture liquid during the 78 h fed-batch cultivation. The pH data are presented as the average of three samples, and OD_600_ data are presented as the average of two samples.

The absorption sensitivities (as the peak area/concentration) for 2BE (1.36 × 10^4^), 2PE (9.69 × 10^3^), and 3HB (3.9 × 10) were determined (Figure S1). It was found that 2-alkenoic acid could be detected with higher sensitivity than 3HB because it is an unsaturated fatty acid with multiple bonds in its structure, which leads to strong UV absorption [32,33,34]. By comparing the absorption sensitivities of 2BE and 2PE, it was found that 2BE can be detected with 1.4-fold better sensitivity than 2PE.

#### 3.2.2. Calibration Curve Results of P(3HB) and P(3HV) Homopolymers

**Figure 4:**
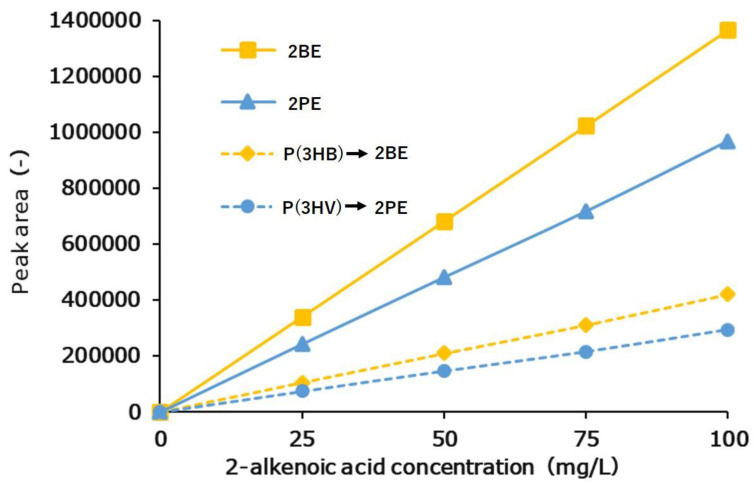
The calibration curves of standard 2-alkenoic acid and 2-alkenoic acid produced by the alkaline degradation of P(3HB) and P(3HV).

The calibration curves of 2BE and 2PE generated by pretreatment of P(3HB) and P(3HV) with alkaline decomposition are shown in Figure S2. Since there is no standard sample for P(3HV), it was produced in this study from *C. violaceum*. From the ratio of the slopes of the calibration curve, the production ratios were determined as α = 3.26 and β = 3.30 (Figure 4). These results indicate that short-chain-length PHA (scl-PHA) can be quantified easily and with high sensitivity by measuring the 2BE and 2PE contents of actual samples using the method proposed here.

The formation of 2BE and 2PE was observed from the alkaline degradation of P(3HB) and P(3HV), respectively, and the prepared calibration curves showed linearity. This indicates that the production ratio α of 2BE from P(3HB) and the production ratio β of 2PE from P(3HV) were constant. On the basis of the ratio of the calibration curve slopes, the production ratios were determined to be α = 3.26 and β = 3.30.

#### 3.2.3. Quantification of Copolymer P(3HB-*co*-3HV)

Copolymer P(3HB-*co*-3HV) standard was purchased from Sigma-Aldrich (containing 12% 3HV), and P(3HB-*co*-3HV) samples were produced in this study using *Bacillus* sp. CYR1. Both the standard and sample P(3HB-*co*-3HV) were pretreated using the alkaline decomposition method, and the resulting 2BE and 2PE contents were analysed via HPLC. For this, different known concentrations of standards were injected (0, 25, 50, 75, 100 mg/L) and then measured. Overall, the measured concentrations (0, 25.6 ± 1.23, 52.9 ± 1.82, 76.5 ± 3.31, 104.8 ± 4.23 mg/L) were very similar to the known concentrations (Table 1), indicating that P(3HB-*co*-3HV) was quantified with high accuracy using the proposed HPLC-based quantification method. The P(3HB-*co*-3HV) standard was known to comprise 88% 3HB and 12% 3HV, but the HPLC analysis in this study showed a concentration 2.35–4.8% higher than the actual value. The impurities in the 2BE (>99% pure) and 2PE (>96% pure) might have contributed to the overestimation of the HV content. Watanabe et al. [26] reported that acid hydrolysis with H_2_SO_4_ at 100 °C converted P(3HB) to 2-butenoic acid and that analysis by HPLC with a UV detector at 210 nm was very convenient, but it is not convenient for other PHAs, because PHA monomers longer than 3HB cannot be converted to the corresponding unsaturated fatty acids. They also conducted alkaline hydrolysis of dried cells in a 96-well assay plate with subsequent HPLC analysis. To produce 2-alkenoic acid, which has strong UV absorption due to its unsaturated bonds, it is necessary to eliminate the α-proton of the 3HA unit. GC and nuclear magnetic resonance (NMR) analyses are the prominent methods for analysing PHA composition. However, as these methods require the use of toxic solvents, they are suitable for the exact quantification of PHAs but are for high-throughput assays. Therefore, the proposed HPLC-based method is preferable.

### 3.3. GC Results

After injecting the 50 mg/L P(3HB-*co*-3HV) standard, a concentration of 47.8 ± 2.05 mg/L was detected by GC analysis, and a concentration of 47.8 ± 2.11 mg/L was detected by HPLC analysis (Table 2). P(3HB-*co*-3HV) samples quantified by both (GC and HPLC) methods showed a concentration 2.2 mg/L lower than the actual concentration of 50 mg/L. This might be due to contaminants present in the P(3HB-*co*-3HV) samples. The GC and HPLC chromatograms of P(3HB-*co*-3HV) are shown in Figure 4. The OD_600_ value and the concentration of P(3HB-*co*-3HV) showed a correlation, proving that P(3HB-*co*-3HV) can be quantified in dried cells without extraction. This result indicates that the alkaline decomposition method proposed in this study can be successfully applied for P(3HB-*co*-3HV) analysis. Satoh et al. (2016) used the alkaline-digestion–HPLC method for the analysis of PHA in activated sludge [31]. By using a reverse-phase column instead of an ion-exchange column, the costs could be decreased by 85%. Also, reverse-phase columns are commonly used in standard HPLC analysis.

A quantitative method using alkaline pretreatment has previously been reported, but the pretreatment takes about 1 h and 15 min [31]. The quantification method in this study enables simple quantification of P(3HV) and P(3HB-*co*-3HV), which are composed of longer molecular chains than P(3HB), and the time required for pretreatment is about 40 min, including pH adjustment and filtration. Therefore, compared to previous studies, the quantitative method in this study is an excellent method that allows simple pretreatment processes and short-time quantification. Although it was not verified in this study, the NaOH concentration during alkaline pretreatment, the temperature of thermal decomposition, and the reaction time lead to a shortening of the pretreatment time. The higher the NaOH concentration, the greater the degree of PHA decomposition, and increasing the temperature could accelerate this effect [35]. Therefore, it is considered possible to further simplify the pretreatment process by optimising the alkaline pretreatment conditions.

## 4. Conclusions

We obtained P(3HV) homopolymer and P(3HB-*co*-3HV) copolymer using two different bacterial strains, *C. violaceum* and *Bacillus* sp. CYR1, respectively. A fast, simple, and inexpensive method to quantify P(3HV) and P(3HB-*co*-3HV) via HPLC with alkaline decomposition was developed. Additionally, the monomer compositions of the polymers as quantified by HPLC were comparable to those obtained via GC analysis. In future work, HPLC-based quantification will be extended to quantify medium-chain-length PHAs.

## Figures and Tables

**Table 1:** Quantification of standard samples of P(3HB-*co*-3HV).

Prepared Sample Concentration (mg/L)	Measured Concentration (mg/L)		
	3HB	3HV	P(3HB-*co*-3HV)
0	0	0	0
25	23.1 ± 1.17	2.5 ± 0.073	25.6 ± 1.23
50	47.8 ± 1.60	5.1 ± 0.24	52.9 ± 1.82
75	69.3 ± 3.01	7.1 ± 0.31	76.5 ± 3.31
100	95.0 ± 3.91	9.9 ± 0.35	104.8 ± 4.23

Standard samples of P(3HB-*co*-3HV) containing P(3HV) (12%) were purchased from SIGMA-ALDRICH. This P(3HB-*co*-3HV) was pretreated via alkaline decomposition and adjusted to different concentrations. Experiments were performed in triplicate (*n* = 3), and the data are presented as the mean ± standard deviation.

**Table 2:** The results of quantitative analysis obtained by HPLC and GC.

	HPLC Method			GC Method		
	3HB	3HV	P(3HB-*co*-3HV)	3HB	3HV	P(3HB-*co*-3HV)
Sample concentration (50 mg/L)	18.3 ± 0.870	29.5 ± 1.32	47.8 ± 2.11	14.7 ± 0.751	33.1 ± 1.31	47.8 ± 2.05

Quantification of P(3HB-*co*-3HV) produced using the *Bacillus* sp. CYR1 strain. Extraction of P(3HB-*co*-3HV) was performed, followed by the method of Reddy et al. [11]. The extracted P(3HB-*co*-3HV) was pretreated via alkaline decomposition, adjusted to the desired concentration (50 mg/L), and analysed via HPLC. In addition, GC analysis with methanolysis pretreatment was performed to compare P(3HB-*co*-3HV) quantitative properties. Experiments were performed in triplicate, and the data are presented as the mean ± standard deviation.

## Data Availability

Not applicable.

## Supplementary Materials

The following supporting information can be downloaded at: https://www.mdpi.com/article/10.3390/bioengineering10050618/s1, Figure S1: Calibration curves of (A) 2BE, (B) 2PE, and (C) 3HB; Figure S2: Calibration curves of (A) 2BE, and (B) 2PE.
