# Supplementary material for: Quantification of the Monomer Compositions of Poly(3-hydroxybutyrate-co-3-hydroxyvalerate) and Poly(3-hydroxyvalerate) by Alkaline Hydrolysis and Using High-Performance Liquid Chromatography
Source: Bioengineering (Basel). 2023 May 20;10(5):618. doi: 10.3390/bioengineering10050618 (PMC10215500; doi:10.3390/bioengineering10050618)
Supplement: Supplementary file 1 [file bioengineering-10-00618-s001.zip › bioengineering-2332387-supplementary.pdf]

Quantification of the monomer compositions of poly (3-hydroxybutyrate-*co*-3-hydroxyvalerate) and poly (3-hydroxyvalerate) by alkaline hydrolysis and using high pressure liquid chromatography

Kyo Saito [1,#], M. Venkateswar Reddy [2,#], Omprakash Sarkar [3], A. Naresh Kumar [4], DuBok Choi [5] and Young-Cheol Chang [1,*]

[1] Course of Chemical and Biological Engineering, Division of Sustainable and Environmental Engineering, Muroran Institute of Technology, 27-1 Mizumoto, Muroran, 050-8585, Japan; 21041026@mmm.muroran-it.ac.jp
[2] Department of Civil and Environmental Engineering, Colorado State University, Fort Collins, Colorado, 80523, USA; mvr_234@yahoo.co.in
[3] Department of Civil, Environmental and Natural Resources Engineering, Luleå Tekniska Universitet, Luleå-97754, Sweden; ommsarkar@gmail.com
[4] Dept of Environmental Science and Tech, University of Maryland, Hyattsville, Maryland, USA; naresh.amradi@gmail.com
[5] Faculty of Advanced Industry Convergence, Chosun University, Kwangju 61452, Korea; choidb@chosun.ac.kr
Kyo Saito and M. Venkateswar Reddy equally contributed to this paper as first author

* Correspondence: ychang@mmm.muroran-it.ac.jp; Tel.: +81-143-46-5757

Abstract: With the growing interest in bioplastics, there is an urgent need to develop rapid analysis methods linked to production technology development. This study focused on the production of a commercially non-available homopolymer, poly(3-hydroxyvalerate) P(3HV), and commercially available copolymer, poly(3-hydroxybutyrate-co-3-hydroxyvalerate) (P(3HB-*co*-3HV)), through fermentation using two different bacterial strains. The bacteria, *Chromobacterium violaceum* and *Bacillus* sp. CYR1, were used to produce P(3HV) and P(3HB-*co*-3HV), respectively. The bacteria *Bacillus* sp. CYR1 produced 415 mg/L of P(3HB-*co*-3HV) when incubated with acetic acid and valeric acid as the carbon sources, whereas the bacteria *C. violaceum* produced 0.198 g of P(3HV)/g dry biomass when incubated with sodium valerate as the carbon sources. Additionally, we developed a fast, simple, and inexpensive method to quantify the P(3HV) and P(3HB-*co*-3HV) using high performance liquid chromatography (HPLC). As alkaline decomposition of P(3HB-*co*-3HV) releases 2-butenoic acid (2BE) and 2-pentenoic acid (2PE), we were able to determine the concentration using HPLC. Moreover, calibration curves were prepared using standard 2BE and 2PE, sample 2BE and 2PE which are produced by alkaline decomposition of poly(3-hydroxybutyrate) and P(3HV), respectively. Finally, the HPLC results obtained from our new method were compared using gas chromatography (GC) analysis.

Keywords: chromatography; crotonic acid; homopolymer; copolymer; polyhydroxyvalerate

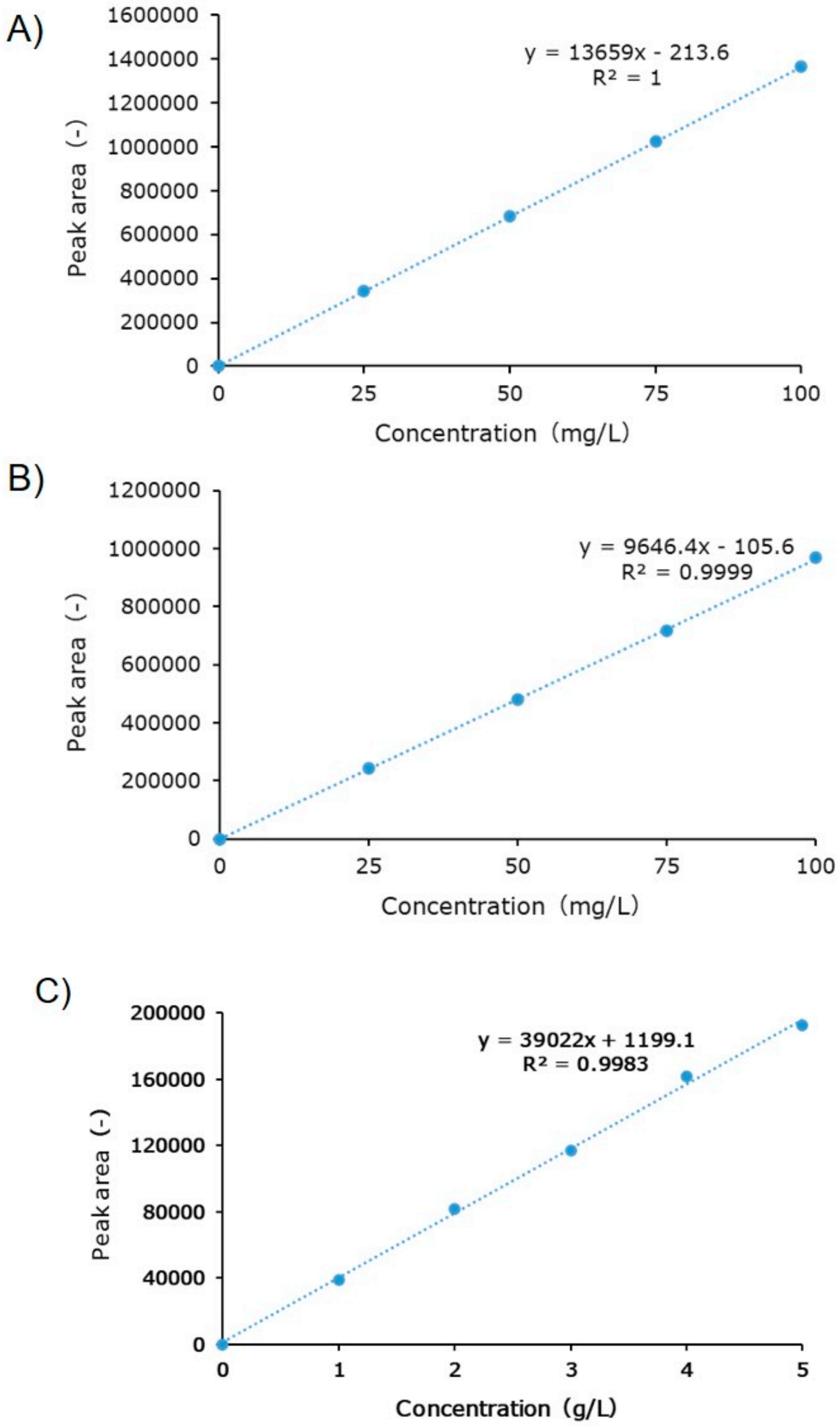

Figure S1. Calibration curves of (A) 2BE, (B) 2PE, and (C) 3HB. The 2BE, 2PE, and 3HB were purchased from Kanto Chemical (Japan), Tokyo Chemical Industry (Japan), and Sigma-Aldrich, respectively.

The absorption sensitivities (as the peak area/concentration) for 2BE (1.36×10^4), 2PE (9.69×10^3), and 3HB (3.9×10) were determined. It was found that 2-alkenoic acid could be detected with higher sensitivity than 3HB because it is an unsaturated fatty acid with multiple bonds in its structure, which leads to strong UV absorption. By comparing the absorption sensitivities of 2BE and 2PE, it was found that 2BE can be detected with 1.4-fold better sensitivity than 2PE.

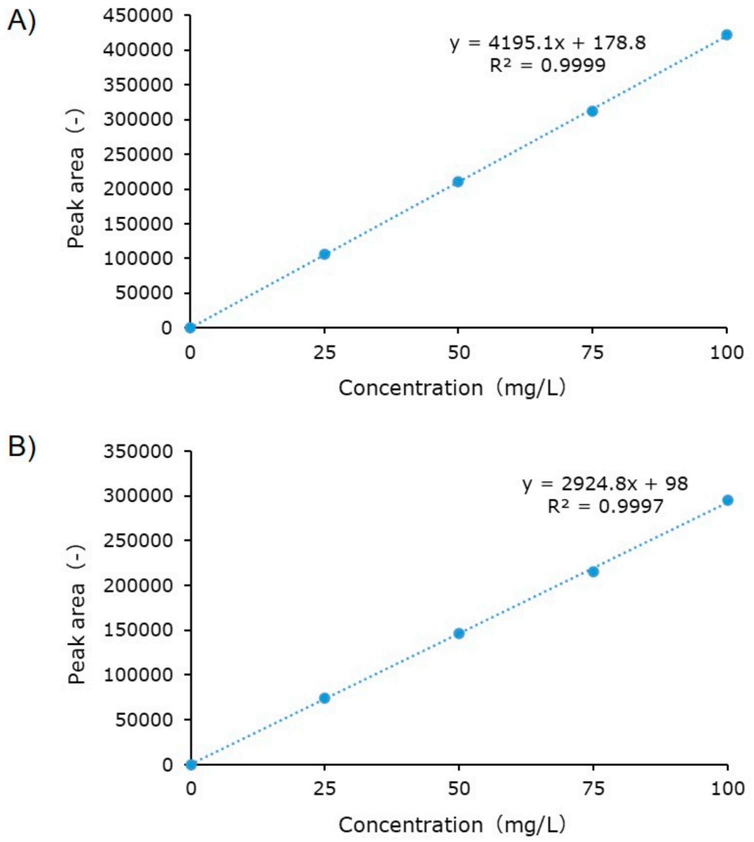

Figure S2. Calibration curves of (A) 2BE, and (B) 2PE. 2BE and 2PE were generated by the pretreatment of P(3HB) and P(3HV) using alkaline decomposition. P(3HB) was purchased from Sigma-Aldrich. Since there is no standard sample for P(3HV), it was produced in this study from *Chromobacterium violaceum*. From the ratio of the slopes of the calibration curve, the production ratios were determined as α =3.26 and β =3.30. These results indicate that short-chain-length PHA (scl-PHA) can be quantified easily and with high sensitivity by measuring the 2BE and 2PE contents of actual samples using the method proposed here.
